# Establishment and Application of the Assessment System on Ecosystem Health for Restored Urban Rivers in North China

**DOI:** 10.3390/ijerph19095619

**Published:** 2022-05-05

**Authors:** Fei Xu, Yonggang Wang, Xu Wang, Dayong Wu, Yuanyuan Wang

**Affiliations:** 1 Beijing Municipal Research Institute of Eco-Environment Protection, National Engineering Research Center for Urban Environmental Pollution Control, Beijing 100037, China; wangyonggang@cee.cn (Y.W.); wangxu@cee.cn (X.W.); wangyuanyuan@cee.cn (Y.W.); 2 Hebei Key Laboratory of Wetland Ecology and Conservation, Hengshui 053000, China; dayongwu@hotmail.com

**Keywords:** river ecosystem, ecosystem health assessment, ecological restoration, indicator system

## Abstract

The study on ecosystem health evaluation for restored urban rivers is of specific significance to improving river health and realizing the adaptive management for urban river ecosystems. Based on the health definition of restored urban rivers in North China, this study attempted to set up a system of alternative indicators on ecosystem health assessment, including water quality, water regime, aquatic organism, riparian environment, and physical morphology. Additionally, a set of health assessment system was proposed, including selection of assessment indexes and determination of assessment criteria and health classes. Taking seventeen typical restored urban rivers in Beijing as the assessment target, the said system was applied in assessing urban river health in 2016 and 2019. As the assessment results indicated, in 2016, the health statuses of 29 percent of urban rivers were ordinary, while 71 percent of urban rivers were somewhat inferior. In 2019, the health state of only one urban river reached “good” level. The health statuses of 88 percent of urban rivers were ordinary, and 6 percent were somewhat inferior in terms of comprehensive health index. In 2019, the health states of rivers improved significantly compared with that of 2016, which indicated that most urban rivers saw marked improvement in ecosystem health after ecological restoration. The health assessment system proposed in the paper not only could be applied to regular evaluation of restored urban rivers in the north but also was suitable for a contrastive health-state analysis between different years prior to or after the restoration. In order to carry out adaptive management of water ecology in urban rivers, the measures of ecological restoration could be adjusted based on the regular health assessment and health weakness analysis.

## 1. Introduction

The birth and development of a city are closely linked to its river system. Most major cities around the world were generally built near one or more rivers. The rivers in the cities could perform the functions of serving residents with water, flood control, shipping, and landscape entertainment but also suffer from the stress of urban development [1,2,3]. The influence of urban development on water ecosystems is mainly manifested as river pollution caused by urban sewage discharge, and the increase of urban water demand occupies a river’s ecological water. At the same time, urban development and construction activities have changed the process of the urban hydrological cycle, and the urban river ecosystem has degraded, and the ecological service function has declined. In recent years, the problems of river ecosystem in cities has drawn extensive public attention. River water shortage, water pollution, and river channelization have led to a decrease of aquatic organism groups and water volume in rivers, change in the community structure, loss of biodiversity, and grave degradation of river ecosystems [4,5,6]. The river ecological degradation has threatened the public interests, which make it increasingly urgent in social needs for improving the quality of rivers’ ecological environment. As the most key carrier of resources and environment, rivers impact existence in the city and constrain city development. Therefore, it is of much urgency to resolve the contradiction between economic development and the urban river ecosystem [7].

The concept of ecosystem health emerged in the 1980s [8,9,10,11,12,13]. Rapport [14] expounded this concept for the first time by arguing that the health of an ecosystem referred to the stability and sustainability of ecosystem, possessing the abilities of maintaining its organization structure, self-adjustment, and restoration from stress and the functions of serving people with sustainable and good ecosystem services. Starting from 1980s, ecosystem health as a metaphor was widely applied in the assessment and management of aquatic ecosystems. The assessment and study work on river ecosystem health started early abroad. Marked progress had been achieved in establishing a set of river ecosystem assessment systems in Australia [15,16], South Africa [17], US [18,19], and the UK [20]. Australian River Assessment Scheme (AusRivAS), River Invertebrate Prediction and Classification System (RIVPACS), Index of Steam Condition (ISC), and Rapid Bioassessment Protocols (RBPs) were all river ecosystem health assessment systems proposed by different countries based on their respective river ecosystem characteristics.

As the concept of ecosystem health was applied to the urban rivers, the assessment and research on urban river health achieved some development in China. Some experts drew upon the assessment system of ISC to set up the system of indexes on urban river health and conduct the assessment. In the early stage, Zhao and Yang [21] applied the theory of river ecosystem in research on urban rivers by defining the health of urban rivers and assessing the river health in the city of Ningbo based on the indicator system including five factors, i.e., water volume, water quality, aquatic organism, physical structure, and riparian zone. Wu et al. [22] established the indicator-based assessment system of urban river health and applied it to river management in Shanghai. The system was composed of five first-tier indexes, including river hydrology, river morphology, riparian zone, physicochemical parameters of water quality, and aquatic organism, as well as seventeen second-tier indexes. Deng et al. [23] carried out a health assessment on the rivers in the city of Li River based on his indicator system of 24 indexes in three aspects, i.e., natural biology, social economy, and landscape environment. Based on the river features in coastal plain city, Ding [24] selected 21 indexes to establish a health assessment system for urban rivers in Yancheng.

There is wide divergence between different regions in terms of urban river characteristics in China. For example, the rivers in the south are abundant in water, while those in the north are generally short of water. Differences also exist in terms of water regime and biological condition. The rivers in the north are characterized by seasonal water, a high degree of exploitation and utilization, large amount of pollutants within, and a lack of ecological water demand, which determines the differences with assessment index and criteria selected for the urban river health between the north and other regions. Meanwhile, a set of river projects for water ecological restoration has been launched in many northern cities. Marked improvement can be seen in terms of water quality, hydrology, and bank landscape in some rivers. However, due to some uncertainty in the ecological restoration [25,26] and the need to evaluating the effect of ecological restoration based on health assessment [27], it is thus necessary to carry out study on the assessment system of ecosystem health for those restored urban rivers in North China, which would provide a certain reference for the future health evaluation of similar types of restored urban rivers.

Considering the above-mentioned aspects, this paper aimed to study the health assessment system for restored urban rivers in North China. Based on the definition of “healthy” urban rivers in North China, the present study attempted to draw upon the widely applied indexes of river health both at home and abroad to set up an alternative indicator system of health assessment for restored urban rivers in North China, including water quality, water regime, aquatic organism, riparian environment, and physical morphology. A set of health assessment system, including selection of assessment indexes and determination of assessment criteria and health classes, was proposed to lay a solid foundation for the regular assessment and adaptive management of the urban river ecosystem based on the case study of 17 typical restored urban rivers in Beijing.

## 2. Methodology

### 2.1. Health Connotation of Restored Urban Rivers in the North

The urban rivers in the north are generally seasonal, featuring high exploitation, large amounts of pollutants, and lack of water for ecological purpose. The restored urban rivers refer to the rivers channelized with the aim of flood prevention many years ago in the urban areas, which are losing the normal hydraulic interaction affected by human interruption and are returning to the state of near-naturalized rivers after the ecological restoration.

In the early stage, the definition of river health focuses on the natural properties of rivers. Karr [28] regarded river ecological integrity as health. Simpson [29] defined river health as the main process of support and maintenance by the river ecosystem so as to return to the previous undisturbed capabilities. The original undisturbed state of rivers was defined as healthy. However, due to severe impact of human activities on urban rivers in the process of city formation and development, it remains difficult to go back to an undisturbed state even after ecological restoration of urban rivers. Furthermore, the urban rivers not only need to sustain the structure and functions of their own ecosystem, but more importantly, they need to offer corresponding functions of ecological service for urban people. Therefore, the characteristics of urban rivers and their natural and social properties should be taken into account when defining the health of restored urban rivers in the north.

The paper draws upon the Australian concept about “healthy working rivers” and the definition of urban river health by Zhao and Yang [21]. Taking into account the natural and social properties of urban rivers and concerning the five factors of river ecosystems and the characteristics of urban rivers in the north, the health connotation of restored urban rivers in the north can be defined as follows: (1) The water quality of rivers is healthy and meets the criteria of corresponding water function. There is no eutrophication in the low-velocity rivers and no risk of pollutant release into the water with relatively clean sediment. (2) The water regime is healthy, with rivers keeping certain water depth and velocity all year round. The ecological water consumption is basically sufficient. (3) The aquatic organisms are healthy. The organisms remain relatively abundant in variety and quantity, with certain biodiversity. (4) The riparian environment is healthy, with the riparian zone normally playing the ecological function of buffer, offering biological habitat, landscaping, and recreation. The rivers can sustain the hydraulic connection without influence on flood control. (5) Finally, the physical morphology is healthy, having a diverse and winding flow state and maintaining certain longitudinal continuity. The ecological function of flood water storage can be performed normally.

### 2.2. Initial Selection of Assessment Indicator System

According to the widely accepted assessment indicator system about river health both at home and abroad as well as the health connotation of restored urban rivers in the north as described above, the study is based on the structural integrity of the river ecosystem and aimed at the normal fulfillment of ecological services. Five aspects of indexes can be initially selected as the alternative indicators of the health assessment on the ecosystem of restored urban rivers in the north, including indexes of water quality, water regime, aquatic organisms, and riparian environment and physical morphology.

(1) Index of Water Quality

The water quality index is selected based on the impact upon water environment quality of rivers by the water body and sediments. In terms of water body and for management of water environment as well as in combination of features of most urban rivers in low-speed flow and general eutrophication, the following indexes were initially selected to indicate the quality of water environment of rivers, including water quality standard rate of water functional area, proportion of water sections on class III and above, as well as the eutrophication index. In general, in the sediments in the urban rivers, there exist some degree of heavy metal and organic contamination. Under certain conditions, the pollutants in the sediment of rivers may be released and contaminate the water. Even if the pollution of the water body is improved, the release of pollutants in the sediment may still pose a potential threat to river water quality. Hence, the sediment pollution index was used to comprehensively characterize the pollution status of heavy metals and nutrients in sediment and further reflected the water environment pollution pressure of urban rivers.

(2) Index of Water Regime

Water regime index selected the intensity of water resource development and exploitation to indirectly represent the impact of human activities upon the water regime of rivers. As for the urban rivers under restoration in the north, whether the river ecological water consumption could be guaranteed directly affects the health of river ecosystem. The guarantee rate of river ecological water consumption was selected to represent the water regime of rivers. The ratio of dry-season runoff volume in the total annual, which could reflect the function of catchment (flood regulation) to replenish and dry up and measure the satisfaction degree of river ecological water demand, was used. When it was difficult to obtain the data of annual average runoff and runoff in the dry season, the alternative index could be evaluated by referring to the current water volume in the normal season. Considering the impact of human control, including flood control and landscaping, upon the restored urban rivers, the natural seasonal variation of water quantity was not significant. The change of water volume in different periods could not reflect the capacity of flood regulation and replenishment. Thus, the water depth and flow velocity were selected to reflect the water regime of rivers.

(3) Index of Aquatic Organisms

As the core component of river ecosystem, the species and quantity of aquatic organisms were important indicators of river ecosystem health. The biological condition index is targeted at the main types of creatures in the ecosystem of restored urban rivers. The indexes about phytoplankton, zooplankton, benthic macro-invertebrates, fish, and macrophytes were selected, and the composite index of biodiversity was also chosen to represent the characteristics of species and a quantity of different aquatic organisms in the river. The proportion of the area of macrophytes to water surface area was considered to reflect the growth and distribution of aquatic plants in rivers. In the process of health assessment, it could be selected according to the situation of the study area and the difficulty of data acquisition.

(4) Index of Riparian Environment

The river corridor plays an important role in purifying water quality, improving the habitat quality, maintaining the biodiversity, and improving the landscaping. Based on ecological corridor principle, the river corridor includes river water body and vegetation zones distributed along the river that differ from the surrounding substrate. The landscape ecological service function of the corridor is mainly experienced and perceived based on the continuous space of riparian vegetation. Riparian zones have many functions, such as providing habitats for animals and plants, filtering, permeating and blocking through vegetation, providing material debris for organisms in the river, preventing soil erosion, preventing wind and fixing sand, regulating flood, and so on [30]. The vegetation coverage of the riparian zone directly affects the fulfillment of above-mentioned functions. With the rapid development of urbanization, human economic activities have made a great deal of artificial transformation of riparian vegetation space, which has destroyed the ecological function structure of the landscape corridor and the natural circulation process of the ecosystem to a great extent [31,32]. For example, when the riparian green space is occupied, the landscape corridor is “broken”, the vegetation ecosystem in some reaches of the river is rapidly degraded, and the ecological service quality is poor. In order to reflect the ecological service functions of the river corridor in the cities, the coverage rate of riparian vegetation was selected to represent the area of the riparian belt starting from the water level of rivers at the normal time and extending 100 m by two sides. The riparian vegetation coverage rate was obtained by calculating the percentage of natural vegetation and artificial vegetation in the total riparian area, which was used to characterize the condition of riparian vegetation coverage.

Due to more focus on flood control and water drainage of urban rivers, some riparian zones were replaced by an excess of hardened or concrete guard wall, causing the loss of ecological function. The percentage of construction land in the riparian zone was used to represent the pressure from the riparian zone placed by urbanized construction land upon the river ecosystems, thus indirectly reflecting the human interruption of the riparian zone.

The riparian zone can play its ecological function effectively only when it meets certain width requirements [33]. Affected by the construction and development activities, the riparian zone of urban rivers generally could not reach the ideal width where all the ecological functions could be normally performed. The riparian width, that is, the width from two sides at the junction of a river and the land in the area where the river influence disappears, was adopted to represent the pressure caused by the occupation of urbanized construction land over the riparian zone towards the river ecosystem and indirectly reflects the human interruption of the riparian zone.

The natural embankment of the riparian zone in the cities was reduced due to the interruption of human activities, such as manual solidification and other engineering construction. Thus, the proportion of natural bank decreased, and the near-natural condition of the riparian zone was poor, which would destroy the horizontal connectivity of riverway and affect the ecological function of flood regulation and storage. The rate of near-natural embankment, that is, the percentage of length of natural and ecologically restored artificial banks in urban rivers to the length of river banks, was selected to represent the connectivity condition between water and land within the riparian zone.

The urban rivers are an important factor of urban ecological balance, and they are the green lifeline of the city. Recreation and beautification of the environment are one of the main functions. Whether the area proportion of the landscape close to the river could meet the needs of residents in the vicinity was the important indicator to reflect whether the ecological functions of rivers could be normally performed. Questionnaire survey can be used to obtain the satisfaction degree of residents regarding the landscape close to the river.

(5) Index of Physical Morphology

River morphology and geomorphology play an important role in maintaining aquatic biodiversity. The physical evolution of the winding section of the river was frequent, and the landforms were diverse, which could provide diversified habitats for aquatic organisms. The sediment, morphology, and connectivity of urban rivers are substantially affected by the urban construction. The physical morphology indicators were adopted to reflect the interruption caused by human activities within the habitat of aquatic organisms in the rivers, and these indicators indirectly represent the health of the river ecosystem. Five indicators were selected to reflect the health of urban rivers from the perspective of physical morphology, including longitudinal connectivity index, river winding rate, degree and influence of river reconstruction, ratio of river bed hardening, and condition of river width change.

The longitudinal connectivity index was used to reflect the human interruption on the longitudinal linkage of rivers. The index was expressed based on the number of dams for every 100 km length of river. The bigger the number, the poorer the longitudinal linkage of the rivers. The winding rate of rivers refers to the ratio of river length to the linear length of rivers so as to reflect the winding condition of rivers. The more winding the river, the more frequent the physical evolution of the curved riverway, the more diverse the geomorphic types, and the more various habitats could be provided. In order to ensure the normal play of flood control and drainage, most of the urban rivers had been channelized. As the curve cut-off engineering might seriously damage the natural morphology of rivers, the winding rate could be adopted to reflect the morphology damage of urban rivers.

The degree and influence of river reconstruction were used to reflect the influence of the loss of ecological function of river physical structure, such as river sediment, morphology, and connectivity, on the surrounding residents. The questionnaire could be adopted to gain the comments of residents about river reconstructions and their impact, such as dam construction, river channelization, and sediment dredging.

The ratio of hardened riverbed was used to represent the influence of human activities on the riverbed and reflect the destruction of the physical habitat of urban rivers. The condition of river width change is defined as the extent to which river width changes during channelization. The two indicators could be obtained from field investigation and estimation.

### 2.3. Indicator System and Weight Identification

The alternative indicator system should be combined with the actual situation of the study area to conduct applicability analysis to establish the final indicator system for health evaluation. When some indexes proved difficult in obtaining data or achieving quantification or even overlapping, the applicability analysis of indexes was needed based on the experts’ opinions to adjust some indexes. Meanwhile, the correlation analysis and principal component analysis could be carried out by using the field monitoring data of the study area to determine a set of assessment indexes that follows the principles of hierarchy, scientific nature, operability, representativeness, and data availability.

The weight of assessment index could be determined by combining subjective and objective methods. Firstly, for the objective weight method, entropy weight method could be applied to preliminarily determine the weight of each assessment index, and then, the weight of each assessment index was adjusted through expert consultation, and finally, the weight coefficient of each assessment index was obtained.

### 2.4. Setting of Assessment Criteria

Based on the health connotation of restored urban rivers in the north and their ecological condition, river health could be divided into five levels from “excellent”, “good”, and “ordinary”, to “somewhat inferior” and “inferior”, respectively, assigned by score threshold “80–100”, “60–80”, “40–60”, “20–40”, and “0–20” at the five levels. For the indexes that were difficult to be expressed quantitatively and accurately, urban rivers with relatively successful ecological restoration were selected as reference points and determined by comparative analysis of field investigation results.

### 2.5. Identification of Health Level

Based on the assigned value and the weight of each health evaluation index, the comprehensive health index (*I_CH_*) of restored urban rivers in the north was calculated based on the following formula.

(1) $$I_{CH}=\overset{n}{\underset{i=1}{\sum }}I_{i}\times W_{i}$$

where *I_CH_* represents the comprehensive health index; *I_i_* is the value of index *i*; and *W_i_* is the weight of the index *i* and can be determined by entropy weight method and expert consultation.

The river health level could be identified in accordance with the health classification standard (Table 1).

## 3. Application of Health Assessment on Restored Urban Rivers

### 3.1. Study Area and Data Sources

**Figure 1:**
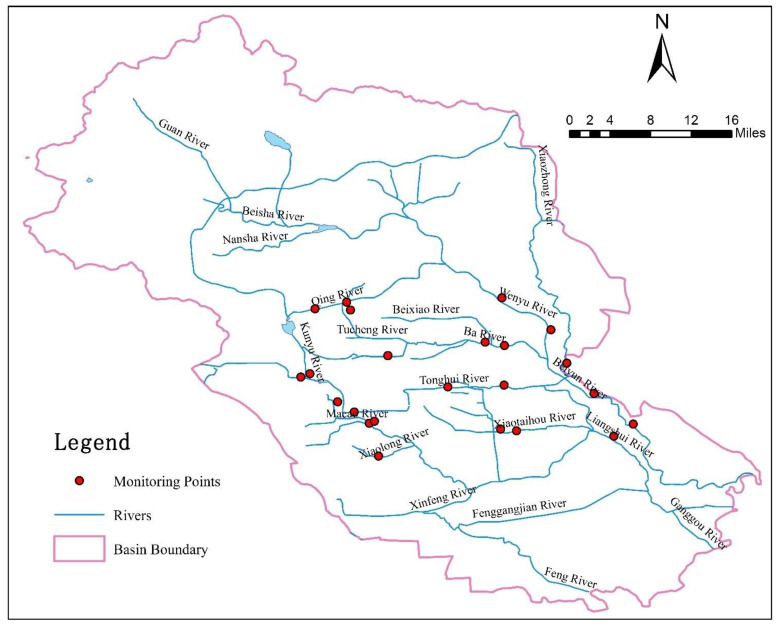
Monitoring points of the typical restored urban rivers in the Beiyun River Basin.

In this study, 17 typical restored urban rivers in the Beiyun River Basin of Beijing were selected as the assessment objects (Figure 1). The main stream of the Beiyun River Basin (Shahe Waterlock to the city boundary) has a total length of 90 km. The Beiyun River Basin is located in the southeast of Beijing, with a watershed of 4348 km^2^, which is the largest water system in the plains of Beijing. The number of rivers in the basin constitute an important part of Beijing’s urban water system, performing the functions of flood control and drainage, ecological landscape, leisure, and entertainment. The Beiyun River Basin has a temperate continental monsoon climate, characterized by cold and dry winters, hot and rainy summers, dry and windy springs, and large temperature changes in winter and summer. In winter, it is mostly northwest wind with an average speed of 3.0–3.5 m per second and a maximum speed of 22.0 m per second. The average annual evaporation on land is 400–500 mm, and the average annual evaporation on water is 1120 mm. The Beiyun River Basin has 14 main streams and primary tributaries and 95 main secondary and tertiary tributaries. The Beiyun River Basin covers 10 districts and 40 towns in Beijing. In 2008, the total population of the basin reached 9.635 million, accounting for more than 70% of the city’s population and more than 80% of its GDP. It is the basin with the largest concentration of population, the most concentrated industries, and the highest level of urbanization in Beijing. In addition, the Beiyun River Basin is an important base to guarantee the supply of grain, vegetables, and fruits in Beijing and an important pillar of Beijing’s agricultural system, which plays a decisive role in the economic development of Beijing.

In the early days, in order to ensure the flood control to the maximum extent, most of the urban rivers were channelized, and the river ecological degradation was quite serious. The water ecological conditions of Beijing’s urban rivers were not optimistic. In recent years, a series of near-natural water ecological restorations had been carried out for urban rivers. The quality of water environment in urban rivers had improved as a whole, but the water quality standard rate of some function areas was still relatively low. Most rivers were faced by a serious shortage of ecological water for consumption and aquatic organism types, mainly pollution-resistant species. There were a certain number of channelized rivers affected by urbanization where the original natural physical forms of channelized rivers were seriously damaged. As Beijing is an international metropolis, the water ecological health status of its urban rivers is one of the important symbols of the city’s development level. It is directly related to the life quality of surrounding residents and has attracted widespread attention from all walks of life. It is an inevitable trend of future development to carry out routine river management by means of river ecological health assessment, so it was necessary to conduct an ecological health assessment for its typical restored urban rivers.

The data used to calculate the assessment indicator of river ecosystem health were mainly obtained based on the field monitoring, data collection, and calculation. The data of typical urban rivers about water quality, water regime, aquatic organisms, riparian zone, and habitat condition were mainly from the on-site water ecological monitoring and survey. Between September and October in 2016 and 2019, respectively, the water quality, hydrology, and aquatic organisms were monitored, and the river connection, riparian environment, and habitat conditions were surveyed at 24 sampling points of 17 typical urban rivers. The monitoring items mainly included 24 water quality indexes, the species and number of phytoplankton and benthic macro-invertebrates and fish, the distribution of waterlocks and dams, the vegetation and width and restoration ratio of riparian zone, and the current status of disturbed aquatic habitats. Water quality indicators were monitored in accordance with the Technical Specifications for Monitoring Surface Water and Sewage (HJ/T91-2002). Aquatic biological monitoring methods referred to the Technical Specification for Aquatic Biological Investigation (DB11/T 1721-2020). Phytoplankton samples were collected using a plexiglass water collector to collect 1000 mL water samples on the surface, fixed with 15 mL Lugo’s solution on site, shaken, and brought back to the laboratory. After standing and concentrating, they were identified and counted; benthic macro-invertebrates samples were collected using the D-type hand net, and the samples were fixed with 75% alcohol and brought back to the laboratory for identification and counting; fish samples were mainly collected by a combination of net hanging method and ground cage method. The samples were identified, weighed, and counted on site. Satisfaction degree of residents regarding the landscape close to the river and degree and influence of river reconstruction were obtained by the method of questionnaire survey. Through the questionnaire survey of the public, we could understand people’s degree of satisfaction with the urban river water environment quality and water ecological landscape and understand whether the urban riparian greening and landscape design could meet people’s needs. Residents along the river and professionals familiar with local conditions were selected as the survey objects. Field investigations were used to conduct a questionnaire survey on the ecological environment of the basin. A total of 2450 questionnaires were sent to the streets and communities where the rivers were located.

### 3.2. Assessment Indicators and Weight

#### 3.2.1. Applicability Analysis of Indexes

In terms of water quality index, and according to the analysis of urban river characteristics and water function zoning in Beijing, most rivers were functionally classified as class IV or below (for landscape water use). The assessment and management of water quality was mainly based on whether it could meet the criteria of function zone. Hence, water quality standard rate of water functional area and eutrophication index were selected. The proportion of water sections in class III and above was not applicable to the actual situation of river management in Beijing. Meanwhile, according to the field monitoring results, the sediments of urban rivers in Beijing were contaminated with heavy metals and organic pollutants to a certain extent, and the sediment pollution index was also selected to reflect the water environment pollution pressure of urban rivers.

In terms of water regime index, the scale of water resources development and utilization intensity index was too large. As for most of urban rivers in Beijing, their riverway had very little base flow, mainly replenished by water transfer or reclaimed water; thus, water collection from the riverway did not exist, so this index was not suitable to represent the hydrological status of urban rivers. Furthermore, it was very hard to obtain the data needed for the index guarantee rate of river ecological water consumption, so the ratio of dry-season runoff volume to the total annual runoff was selected to reflect the satisfaction degree of river ecological water demand. Meanwhile, the water depth and flow velocity were also selected to represent hydrological condition of urban rivers.

In terms of biological index, as a primary producer, the status of phytoplankton could rapidly and directly reflect the health condition of urban rivers. Therefore, the comprehensive index of phytoplankton diversity was selected. Macrophytes were relatively less distributed in urban rivers, and the data acquisition was difficult. Hence, the area ratio of large aquatic plants was not considered. Composite index of benthic macro-invertebrates diversity and composite index of fish diversity were selected to reflect the biological condition of urban rivers.

In terms of riparian environment index, the width of the riparian zone is still in the theoretical study stage. Scholars and experts from various countries have put forward the requirements for the width of riparian zone under different ecological functions, but there is to date no universal standard. Since the ecological functions of urban rivers has not yet been clearly defined, it was difficult to determine the grading criteria for the width of the riparian zone, and thus, this index was not considered here. The riparian vegetation coverage rate, percentage of construction land, proportion of near-natural embankment, and degree of satisfaction residents regarding the landscape close to the river were selected to reflect the riparian condition of urban rivers.

In terms of physical morphology index, most of the rivers in Beijing have been renovated by channelization. Some rivers that were ecologically restored only underwent riparian restoration. Most riverbeds basically remained hardened to prevent infiltration of river water, and a few hardened riverbeds had been removed, but some rivers were so deep that the condition of riverbed hardening could not be identified. Therefore, the ratio of hardened riverbed was not considered. Longitudinal connectivity index, river winding rate, degree and influence of river reconstruction, and condition of river width change were selected to reflect the physical morphology condition of urban rivers.

#### 3.2.2. Statistical Analysis and Determination of Indexes

Through the preliminary screening of the evaluation index system and the applicability analysis of the indicators by referring to the opinions of experts in related fields, the evaluation index system of restored urban river ecosystem health in Beijing was obtained, which included 16 indicators concerning the five elements of water quality, water regime, aquatic organisms, riparian environment, and physical morphology. In order to avoid overlapping between indicators, the indexes under assessment were further analyzed and selected based on applied correlation analysis and principal component analysis.

**(1)**  **Correlation analysis** 

The IBM SPSS Statistics 22 software (Statistical Package for Social Science Company, Chicago, IL, USA) was used for analysis of assessment indexes. According to the Pearson correlation coefficient of all indicators, most indicators did not have significant correlation. Only a few indicators, such as riparian vegetation coverage rate and percentage of construction land in the riparian zone, condition of river width change, and river winding rate, had a certain correlation, with Pearson correlation coefficient greater than 0.6.

The correlation analysis results of the above indicators showed that, in spite of certain correlation between the values of these indicators, the Pearson correlation coefficient was basically below 0.6. Only a few indexes had relatively high correlation coefficients, such as riparian vegetation coverage rate and percentage of construction land in the riparian zone, condition of river width change, and river winding rate. Therefore, it was necessary to further analyze the importance of each index to health representation for selection.

**(2)**  **Principal component analysis** 

According to the results of correlation analysis, when two indicators were highly correlated, it was difficult to decide which one to delete. Therefore, the statistical method- Principal Component Analysis (PCA) was used to identify the importance of each index in the assessment indicator system to river health representation. Through the method, the principal components that could cover the overall river health index system were determined, and the contribution of each index to the principal components was analyzed to achieve further selection of assessment index. Among the two related indicators, the one with less contribution would be deleted in the index selection process. The IBM SPSS Statistics 22 software was used to conduct principal component analysis of the assessment indicators. The selection results showed that the cumulative percentage of the first seven principal components reached 91.39%. The selection of these seven principal components met the requirements about the amount of information. From the component matrix, the load size of each assessment index on each principal component could be seen. The greater the load, the greater the correlation coefficient between the index and the principal component. By comparing the correlation coefficients, the indexes covered by each principal component could be determined (Table 2).

According to the corresponding evaluation indexes of each principal component, it could be seen that the 16 indexes used for statistical analysis corresponded to the seven principal components extracted, respectively. According to the results of correlation analysis, the indexes whose Pearson correlation coefficient was greater than 0.6 were the riparian vegetation coverage rate and percentage of construction land in the riparian zone, condition of river width change, and river winding rate. Considering the results of principal component analysis, the riparian vegetation coverage rate was relatively more important than the percentage of construction land in the riparian zone, and the river winding rate was relatively more important than the condition of river width change. Therefore, the two indexes of the percentage of construction land in the riparian zone and the condition of river width change were deleted.

**(3)**  **Determination of indexes** 

After initial selection, applicability analysis, statistical analysis, and expert consultancy, the health assessment index system of Beijing’s restored urban river ecosystem was finally determined, including 14 indicators concerning the five aspects of water quality, water regime, aquatic organisms, riparian environment, and physical morphology (Table 3).

#### 3.2.3. Identification of Index Weight

The weight of each assessment index was determined by combining subjective and objective methods. The entropy method was used first to initially determine the weight of each assessment index [34]. In order to ensure the rationality of the weight coefficients determined, the expert consultation method was applied to appropriately adjust the above-mentioned weight. First, according to the principles of urban rivers’ health assessment indicator system and the principle of analytic hierarchy process, the expert consultation form about the element layer and the index layer was designed. Then, the questionnaire was issued to experts from the universities and scientific research institutes in the research fields of environmental science, hydrology, ecology, aquatic biology, and urban planning, etc., and was collected for data sorting. The average weight obtained from the expert consultation was calculated to determine the reference weight of each index. According to the index reference weight obtained by the expert consultation method, the weight coefficient calculated based on the entropy weight method was appropriately adjusted to further determine the final weight of each assessment index of the restored urban river ecosystem health.

### 3.3. Assessment Criteria

Assessment criteria of the restored urban river ecosystem health was identified mainly by referred to the Technical Guide for Watershed Ecological Health Assessment proposed by the original environmental protection in China. Among the assessment indicators, the sediment pollution index was integrated by selecting the Nemerow comprehensive index, which reflects the heavy metal pollution and the organic index that could reflect the nutrient pollution. The grading criteria of each index was determined based on the field monitoring results. The identification of degree and influence of river reconstruction upon the indicator evaluation criteria referred to the grading interpretation about the riverway change and sediment characteristics in rapid assessment sheet of river habitats. According to the indicator assessment criteria about degree of satisfaction of residents with the landscape close to the river based on the analysis results of questionnaire, the demands and satisfaction rate of residents surrounding rivers for the riparian environment were identified under different levels (Table 4).

### 3.4. Assessment Results and Analysis

Taking 17 restored urban rivers of Beijing as the target, and based on the water ecological monitoring in 2016 and 2019 and the assigned value and weight of each health assessment index, the comprehensive health index about rivers was obtained by applying the calculation formula, and the health level was identified based on the health grading criteria. The health assessment results of typical restored urban rivers were listed in Table 5.

The health assessment results indicated that in 2016, 5 of the 17 urban rivers had a comprehensive health index above 40, which was in the ordinary grade. Among them, the Northern Hucheng River and Wenyu River were relatively healthy. The comprehensive health index of the remaining 12 rivers was between 20 and 40, which was in the somewhat inferior grade, among which the Xiaolong River, Xiaotaihou River, diversion canal of Yongding River, and Mazao River were in relatively poor health. In 2019, among the 17 urban rivers, the comprehensive health index of Lianhua River reached 62.4, denoting a good level. Among the other 16 rivers, 15 rivers had a comprehensive health index of over 40, which was of the ordinary grade. Among them, the canal of Tonghui River, Tonghui River, South Hucheng River, and Northern Hucheng River were relatively healthy. Only the Xiaozhong River had a relatively poor comprehensive health index between 30 and 40.

In order to enable managers to take effective management measures based on the results of the health assessment, we recommended continuing to monitor and protect the current status of “excellent” rivers. In the case of “good” and “ordinary” rivers, appropriate improvements can be made according to existing weaknesses in health; large-scale ecological restoration should be carried out for “somewhat inferior” and “inferior” rivers. According to assessment results in 2019, most rivers were of ordinary grade. Lack of ecological water demand, low aquatic biodiversity, and physical form damage were the main causes of river health degradation. It was suggested that appropriate restoration measures should be taken according to the existing problems.

**Figure 2:**
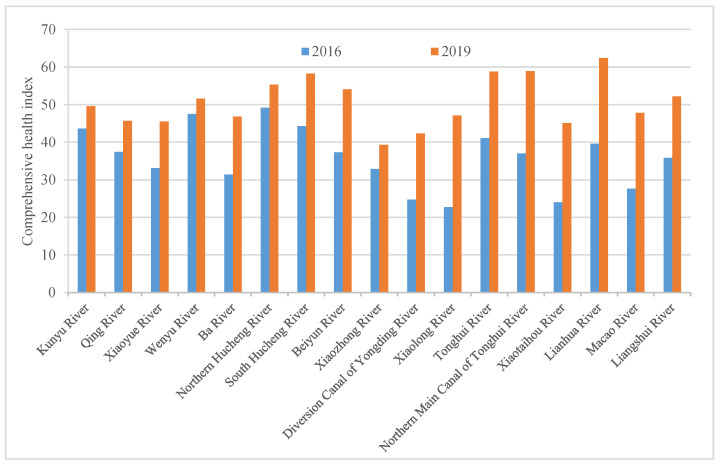
Health comparison of typical restored urban rivers in 2016 and 2019.

For restored urban rivers with more manual control, changes of their health status could reflect the effectiveness of management measures. Thus, we used comparative analysis of health in different years to grasp the extent of health changes for various rivers. According to the comparative analysis results, the management measures adopted for rivers with better health could be used as a reference. To compare the health of 17 rivers in 2016 and 2019, the comprehensive health index of rivers in 2019 increased as compared to that in 2016 (Figure 2). The Xiaolong River, Xiaotaihou River, Macao River, diversion canal of Yongding River, canal of Tonghui River, and Lianhua River recorded a growth rate of more than 50%. About 29% of 17 rivers remained at the ordinary level. A total of 59% of rivers were upgraded from the somewhat inferior level to the ordinary level. The health level of the Lianhua River was improved from the ordinary level to the good level, indicating the improvement in river ecosystem health.

## 4. Discussion

The ecological restoration of rivers played a key role in improving the health condition of the degraded river ecosystem, but the restoration effect might not be reflected in the short term. The tracking monitoring and the effect analysis of river ecosystems after ecological restoration were conducted in different countries. In 1994, two secondary channels were built to restore the habitat of the water community in the Rhine. A five-year post-project monitoring and analysis plan was set up, including monitoring and analyzing the aquatic macroinvertebrates, fish, and wading birds [35]. In 2001, Project River Recovery (PRR) was adopted for effect analysis to recover the habitat of rivers and wetland with gravel beds in New Zealand. By analyzing the habitat improvement of the PRR in the past 10 years, the advantages of the project and the areas needing improvement were obtained. [36]. In 2012, U.S. experts applied a set of methods to analyze the impact of ecological restoration on the rivers in the central region of New York, indicating that the restoration project had achieved certain effects. [37]. Since 2016, a series of river ecological restoration projects have been launched in Beijing, including water replenishment through the South-to-North Water Diversion Project, improvement of river hydrological cycle by using water conservancy facilities, and creation of habitats, which significantly improved the water quality, aquatic life, and riparian landscape of some rivers. It could be seen from the comparison of health assessment results between 2016 and 2019 that noticeable improvement had been achieved in the water ecological health of 65 percent of the rivers under assessment. Hence, as for the urban rivers under restoration, it was quite necessary to properly optimize the restoration measures and carry out water ecological monitoring and health evaluation regularly in order to verify the effect of water ecological restoration.

**Figure 3:**
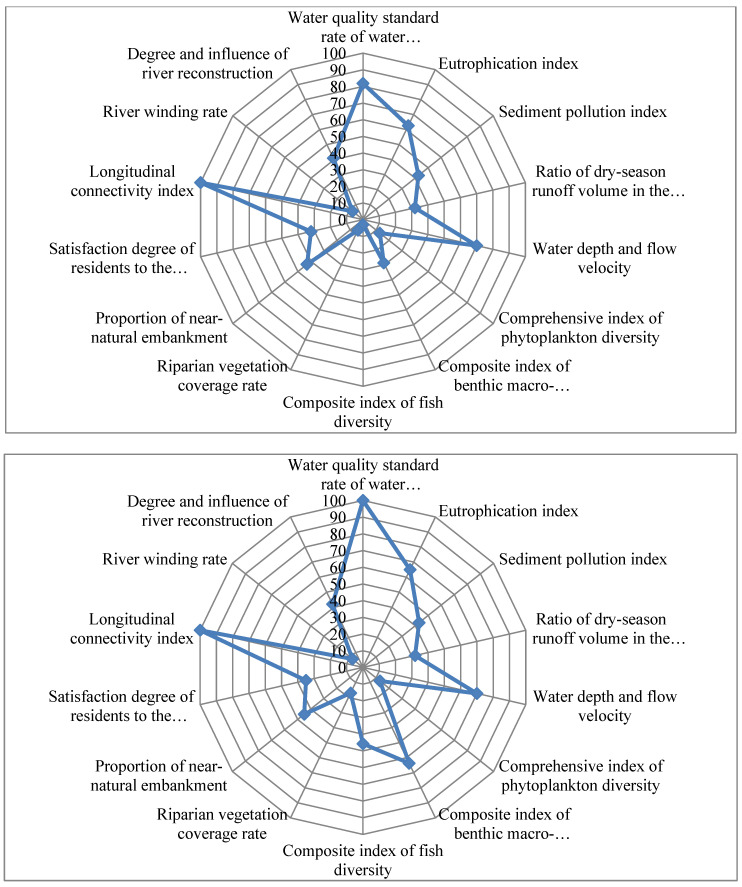
Score distribution of different indexes of the Lianhua River in 2016 and 2019.

The health assessment system of restored urban rivers in the north established in this paper could be used to evaluate the health condition of these rivers prior to or after the restoration or between different years after the restoration for comparative analysis. Based on the above-mentioned assessment results, the radar map was adopted for the contrastive analysis over each index of the Lianhua River, which has seen much-improved health in the past two years. Radar map was a graphical method of displaying multivariable data in the form of a two-dimensional diagram of multiple quantitative variables. The radar map could intuitively reflect the numerical gap of each variable and can be used to compare the scores of each index in the process of health evaluation to determine the shortcomings of the river’s health status and the improvement effects of indicators after water ecological restoration. According to the scores of each index for the Lianhua River in 2016 and 2019 (Figure 3), the indexes of physical morphology, riparian environment, and aquatic organisms constituted the major weaknesses of the river’s health. In comparison to 2016, some indicators of water quality, aquatic organisms, and riparian environment improved in 2019 to varying degrees. The water quality standard rate of water functional area was upgraded from 81.82 to 100, the score of composite index of benthic macro-invertebrates diversity increased from 28.8 to 63.7, and the composite index of fish diversity increased from 3 to 45.7, indicating that noticeable improvement was achieved in terms of water quality and aquatic life. The score for riparian vegetation coverage rate rose from 7 to 17, indicating that the riparian environment also improved to a certain extent. Considering the requirements of urban construction and flood control, the Lianhua River was channelized in the early stage when the surrounding riparian zone was occupied, and the ecological function of the river was seriously degraded. Based on the evaluation data, the Lianhua River had been ecologically restored in recent years, with water quality and aquatic organisms being significantly improved. However, its improvement in the water regime, physical morphology, and riparian environment was quite limited. Therefore, in the future, relevant ecological restoration measures should be taken targeting the current health weaknesses to realize the adaptive management of the river ecosystem.

Water quality is an important part of river ecosystems. Whether the water quality can meet the standard is a key factor reflecting ecosystem health status. Through analyzing the data of water quality standard rate of water functional area, we found that in 2016, the average index was 32.6, and 29.4% of rivers were rated above “good” level. In 2019, the average index was 84, and 88.2% of rivers were rated above “good” level. The overall trend of water quality was consistent with that of health status. However, according to our previous research, the use of a single water quality index in environmental management was not enough to reflect the structure and function of the ecosystem. In 2019, 41 percent of rivers scored perfect for water quality but were only in ordinary health, which indicated that it was reasonable to use multiple types of indicators to comprehensively represent health status.

Compared with previous studies on urban river health evaluation, this evaluation index system was relatively flexible and could be selected according to measured data, which avoids the applicability problem of the previous fixed-index system for different study areas and is more conducive to the promotion and application of the evaluation system for restored urban rivers in North China.

## 5. Conclusions

On the basis of the health definition of restored urban rivers in northern China, this study constructed an alternative assessment indicator system of ecosystem health of restored urban rivers in the north based on the structural integrity of the river ecosystem and aiming at the normal functioning of ecological services, including water quality, water regime, aquatic organism, riparian environment, and physical morphology. A health assessment system was proposed, including evaluation index selection, evaluation criteria, and health grade determination, and applied to 17 typical restored urban rivers in Beijing. Based on the water ecological monitoring data in the study area, 14 health evaluation indexes were determined after applicability analysis and statistical analysis. The assignment criteria for five assessment levels of each index, namely “excellent, good, ordinary, somewhat inferior, and inferior”, were identified. According to the health assessment results of the restored urban rivers in 2016 and 2019, the overall health of Beijing’s typical restored urban rivers in 2016 was at a somewhat inferior level, and the overall health status was degraded more severely. However, after a series of water ecological restoration projects, in 2019, the overall ecological health of river waters was at an ordinary level, with the health status significantly improved. This assessment system was not only suitable for the regular evaluation of the restored urban rivers in the north to obtain the change of health state but also for the comparative analysis of the health status before and after the restoration to assess the effect of ecological restoration. Through the evaluation of health status, the existing health problems were identified, and recommendations for different health levels were proposed, which could provide a powerful starting point for environmental management departments to further improve the river water ecological health in the future and provide an important foundation for the realization of adaptive management of urban rivers.

## Figures and Tables

**Table 1:** Health classification of restored urban rivers in the north.

Health	Excellent	Good	Ordinary	Somewhat Inferior	Inferior
Comprehensive health index (*I_CH_*)	*I_CH_* ≥ 80	60 ≤ *I_CH_* < 80	40 ≤ *I_CH_* < 60	20 ≤ *I_CH_* < 40	*I_CH_* < 20

**Table 2:** Corresponding assessment indexes of each principal component.

Principal Components	Assessment Indexes
1st principal component	Satisfaction degree of residents with the landscape close to the river, riparian vegetation coverage rate, degree and influence of river reconstruction, proportion of near-natural embankment
2nd principal component	Ratio of dry-season runoff volume to the total annual runoff, water quality standard rate of water functional area, eutrophication index
3rd principal component	River winding rate, sediment pollution index, comprehensive index of phytoplankton diversity
4th principal component	Longitudinal connectivity index, composite index of benthic macro-invertebrates diversity
5th principal component	Percentage of construction land, condition of river width change
6th principal component	Composite index of fish diversity
7th principal component	Water depth and flow velocity

**Table 3:** Health assessment indicators of restored urban rivers in Beijing.

Destination Layer	Element Layer	Indicator Layer	Indicator Interpretation and Calculation Method
Health assessment indicators of restored urban rivers in Beijing	Water quality	Water quality standard rate of water functional area	Water quality standard rate of water functional area (%), referred to the percentage of the months in which water quality reached the standard of water functional zone in the evaluation months, represented the water environment of urban rivers.
		Eutrophication index	The eutrophication index was calculated in reference to the Regulations of Evaluation Methods and Classification on Lake (Reservoir) Eutrophication ((2001) No. 090). It was used to represent the eutrophication status of urban rivers.
		Sediment pollution index	Nemerow composite index (P), reflecting the pollution of heavy metal and organic index about pollution of nutritive salt (OI), was selected to integrate the index of sediment pollution.
	Water regime	Ratio of dry-season runoff volume to the total annual runoff	Field investigation could be launched for situation judgement over the riverway water volume in normal period.
		Water depth and flow velocity	The water depth and flow velocity were selected to reflect the water volume of manually controlled urban rivers.
	Aquatic organisms	Comprehensive index of phytoplankton diversity	Three indicators were selected for comprehensive evaluation, including the number of algae taxa, the Shannon–Wiener diversity index of algae, and the Berger–Parker dominance index of algae. The indicators were standardized first, and then, the arithmetic mean sum of the three indicators was calculated.
		Composite index of benthic macro-invertebrates diversity	Two indicators were selected including the number of benthic macro-invertebrates taxa and the Berger–Parker dominance index of benthic macro-invertebrates. The index integration method was the same as that of phytoplankton.
		Composite index of fish diversity	Three indicators were selected, including the number of fish taxa, the Shannon–Wiener diversity index of fish, and the Berger–Parker dominance index of fish (D). The index integration method was the same as that of phytoplankton.
	Riparian environment	Riparian vegetation coverage rate	Taking the river water level in the normal period as the starting boundary and extending 100 m on both sides as the riparian zone, the percentage of the areas of natural and artificial vegetation to the total area of riparian zone was the riparian vegetation coverage rate.
		Proportion of near-natural embankment	The percentage of the length of natural banks and ecologically restored artificial banks in urban rivers to the length of river banks.
		Satisfaction degree of residents with the landscape close to the river	The questionnaire was adopted to obtain data about residents’ degree of satisfaction with riparian leisure and recreational functions.
	Physical morphology	Longitudinal connectivity index	The index could be represented by the number of dams per 100 km of river length. The larger the number, the worse the longitudinal connectivity of the river.
		River winding rate	The index referred to the ratio of river length to the linear length of rivers, reflecting the winding state of the river.
		Degree and influence of river reconstruction	Used to reflect the ecological function loss of physical structures caused by urban construction activities. Through the questionnaire, the opinions of residents about river reconstruction were obtained.

**Table 4:** Grading criteria of health assessment indexes of restored urban rivers in Beijing.

Index			Grading Criteria and Value Assignment				
			Excellent	Good	Ordinary	Somewhat Inferior	Inferior
			80 ≤ N < 100	60 ≤ N < 80	40 ≤ N < 60	20 ≤ N < 40	0 ≤ N < 20
Water quality	Water quality standard rate of water functional area (%)		80 ≤ N < 100	60 ≤ N < 80	40 ≤ N < 60	20 ≤ N < 40	0 ≤ N < 20
	Eutrophication index		0 ≤ N < 30	30 ≤ N < 50	50 ≤ N < 60	60 ≤ N < 70	70 ≤ N < 100
	Sediment pollution index	Nemerow composite index	0 ≤ N < 1	1 ≤ N < 2.5	2.5 ≤ N < 4.5	4.5 ≤ N < 7	7 ≤ N < 10; N ≥ 10, value assigned = 0
		Organic index	0 ≤ N < 0.05	0.05 ≤ N < 0.15	0.15 ≤ N < 0.2	0.2 ≤ N < 0.5	0.5 ≤ N < 2; N ≥ 2, value assigned = 0
Water regime	Ratio of dry-season runoff volume to the total annual runoff ①		1.3 ≤ N < 2	1.1 ≤ N < 1.3	0.9 ≤ N < 1.1	0.7 ≤ N < 0.9	0.4 ≤ N < 0.7; N ≥ 2 or N < 0.4, value assigned = 0
			Completely submerged	Submerged greater than 75%	Submerged 50–75%	Submerged 25–50%	Some waterway dried
	Water depth (m)		1.2 ≤ N < 4	1 ≤ N < 1.2	0.8 ≤ N < 1	0.6 ≤ N < 0.8	0 ≤ N < 0.6
	Flow velocity (m/s)		0.5 ≤ N < 1	0.3 ≤ N < 0.5	0.1 ≤ N < 0.3	0.05 ≤ N < 0.1	0 ≤ N < 0.05
Aquatic organisms	Comprehensive index of phytoplankton diversity		0.8 ≤ N < 1	0.6 ≤ N < 0.8	0.4 ≤ N < 0.6	0.2 ≤ N < 0.4	0 ≤ N < 0.2
	Composite index of benthic macro-invertebrates diversity		0.8 ≤ N < 1	0.6 ≤ N < 0.8	0.4 ≤ N < 0.6	0.2 ≤ N < 0.4	0 ≤ N < 0.2
	Composite index of fish diversity		0.8 ≤ N < 1	0.6 ≤ N < 0.8	0.4 ≤ N < 0.6	0.2 ≤ N < 0.4	0 ≤ N < 0.2
Riparian Environment	Riparian vegetation coverage rate		80 ≤ N < 100	60 ≤ N < 80	40 ≤ N < 60	20 ≤ N < 40	0 ≤ N < 20
	Proportion of near-natural embankment		80 ≤ N < 100	60 ≤ N < 80	40 ≤ N < 60	20 ≤ N < 40	0 ≤ N < 20
	Satisfaction degree of residents with the landscape close to the river ②		Adequate water-friendly area; fully meets the demands	Quite large percentage of water-friendly area, basically meeting the demands	Some percentage of water-friendly area, with design weakness, but sufficient to meet the demands	Lack of water-friendly environment and failure to meet the demand	No water-friendly environment in general
Physical morphology	Longitudinal connectivity index (Number of dams/100 km)		0 ≤ N < 1	1 ≤ N < 3	3 ≤ N < 5	5 ≤ N < 10	10 ≤ N < 20; N ≥ 20 value assigned = 0
	River winding rate		3.4 ≤ N < 4	2.8 ≤ N < 3.4	2.2 ≤ N < 2.8	1.6 ≤ N < 2.2	1 ≤ N < 1.6
	Degree and influence of river reconstruction ③		No influence	A slight influence	General influence	Great influence	Serious influence

① This index grading criteria was measured by the proportion of the low banks covered by water. ② This index grading criteria was measured by the proportion of the water-friendly area and whether it had rational design and met the demands of surrounding residents. ③ No influence: The river way maintained a natural pattern by means of flexible revetments composed of wooden stakes, ripraps, and aquatic plants. There was a large quantity of bottom sediment suitable for biological survival; A slight influence: At a small amount of channelization, the revetment was composed of concrete blocks with pores inside, stone cages, and plant materials. There was a certain amount of bottom sediment suitable for biological survival; General influence: There was a certain degree of channelization, and part of the revetment was composed of gabions, porous concrete blocks, and plants. There was a small amount of bottom sediment suitable for biological survival; Great influence: Entirely composed of dry masonry for the revetment, and sediment was often disturbed or removed; Serious influence: The vertical revetment was made of concrete panels. Most of the river section was dredged, with almost no bottom sediment suitable for living things.

**Table 5:** Health assessment results of typical restored urban rivers in Beijing.

Type	Rivers under Assessment	Comprehensive Health Index in 2016	Health Level in 2016	Comprehensive Health Index in 2019	Health Level in 2019
Restored urban rivers	Kunyu River	43.6	Ordinary	49.6	Ordinary
	Qing River	37.4	Somewhat inferior	45.7	Ordinary
	Xiaoyue River	33.1	Somewhat inferior	45.5	Ordinary
	Wenyu River	47.5	Ordinary	51.6	Ordinary
	Ba River	31.4	Somewhat inferior	46.8	Ordinary
	Northern Hucheng River	49.2	Ordinary	55.3	Ordinary
	South Hucheng River	44.3	Ordinary	58.3	Ordinary
	Beiyun River	37.3	Somewhat inferior	54.1	Ordinary
	Xiaozhong River	32.9	Somewhat inferior	39.3	Somewhat inferior
	Diversion Canal of Yongding River	24.7	Somewhat inferior	42.3	Ordinary
	Xiaolong River	22.7	Somewhat inferior	47.1	Ordinary
	Tonghui River	41.1	Ordinary	58.8	Ordinary
	Northern Main Canal of Tonghui River	37.0	Somewhat inferior	58.9	Ordinary
	Xiaotaihou River	24.0	Somewhat inferior	45.1	Ordinary
	Lianhua River	39.6	Somewhat inferior	62.4	Good
	Macao River	27.6	Somewhat inferior	47.8	Ordinary
	Liangshui River	35.8	Somewhat inferior	52.2	Ordinary

## Data Availability

The data used in the study had been obtain from field monitoring. The study did not report any data.
